# Pyogenic liver abscess caused by trans-gastric migration of a toothpick managed with laparoscopic left lateral sectionectomy and gastric perforation repair: a case report

**DOI:** 10.3389/fsurg.2026.1799606

**Published:** 2026-03-19

**Authors:** Zhigao Yuan, Xingfei Li, Jiye Zhu, Jie Gao

**Affiliations:** Beijing Key Surgical Basic Research Laboratory of Liver Cirrhosis and Liver Cancer, Department of Hepatobiliary Surgery, Peking University People’s Hospital, Beijing, China

**Keywords:** gastric perforation, laparoscopy, migrated foreign body, pyogenic liver abscess, source control, toothpick

## Abstract

**Background:**

Pyogenic liver abscess caused by migration of an ingested toothpick through occult gastrointestinal perforation is rare and may be missed.

**Case presentation:**

A 47-year-old woman presented with 2 weeks of fever and epigastric pain. Laboratory tests revealed leukocytosis (13.3 × 10^9/L) and elevated C-reactive protein (45.4 mg/L) with normal procalcitonin (0.03 ng/mL). Non-contrast CT demonstrated an abscess-like lesion in the left hepatic lobe with an intralesional linear hyperdense focus, suggesting a foreign body. After empirical broad-spectrum antibiotics, laparoscopic left lateral sectionectomy with gastric perforation repair was performed. An intact toothpick was retrieved from the abscess cavity, confirming the etiology. The postoperative course was uneventful. This case highlights the diagnostic value of a linear hyperdense focus within a left-lobe hepatic abscess on CT for suspecting foreign body migration and guiding timely source control.

**Conclusion:**

A linear hyperdense focus within a hepatic abscess on CT should raise suspicion for migrated gastrointestinal foreign body. Definitive cure requires prompt source control, including foreign body removal and repair of the perforation tract.

## Introduction

Pyogenic liver abscess (PLA) is a frequent and potentially life-threatening intra-abdominal infection. Common etiologies include biliary tract infection, portal venous seeding, and contiguous spread from adjacent infectious foci. In contrast, PLA secondary to gastrointestinal perforation by an ingested foreign body is uncommon, reported to account for <1% of all liver abscesses (1–3). Because clinical manifestations are often nonspecific and patients may have received antibiotics before presentation, these cases are easily misinterpreted as typical PLA or even as a hepatic space-occupying lesion (3).

Toothpicks are among the more frequently implicated sharp foreign bodies. Notably, most patients do not recall foreign-body ingestion, and perforation can be occult, making preoperative diagnosis challenging (4, 5). Early recognition is clinically important because definitive management hinges on prompt source control—removal of the foreign body and treatment of the perforation tract—rather than antimicrobial therapy alone.

Here, we report a rare case of toothpick-associated PLA caused by transgastric migration into the liver, managed with laparoscopic left lateral sectionectomy and concomitant gastric perforation repair. We highlight key diagnostic clues and the operative strategy, and this report is prepared in accordance with the CARE guidelines for case reports.

## Patient information and history

A 47-year-old woman presented with a 2-week history of fever (peak 38.6 °C) and postprandial upper abdominal pain/distension, maximal over the hepatic region. She denied chills, jaundice, pruritus, or respiratory symptoms. At a local hospital, leukocytosis and elevated C-reactive protein were reported, and her symptoms partially improved after empirical ceftazidime. She had no significant medical history, no viral hepatitis, and no known allergies. Notably, she did not report or recall accidental foreign-body ingestion, and no psychiatric illness or eating disorder history was documented.

## Physical examination

On admission, she was hemodynamically stable with low-grade fever (37.8 °C). No jaundice or peripheral lymphadenopathy was noted, and cardiopulmonary examination was unremarkable. Abdominal examination revealed upper abdominal tenderness with positive hepatic percussion pain, without rebound tenderness; Murphy's sign was negative. Bowel sounds were mildly decreased.

## Diagnostic assessment

**Figure 1:**
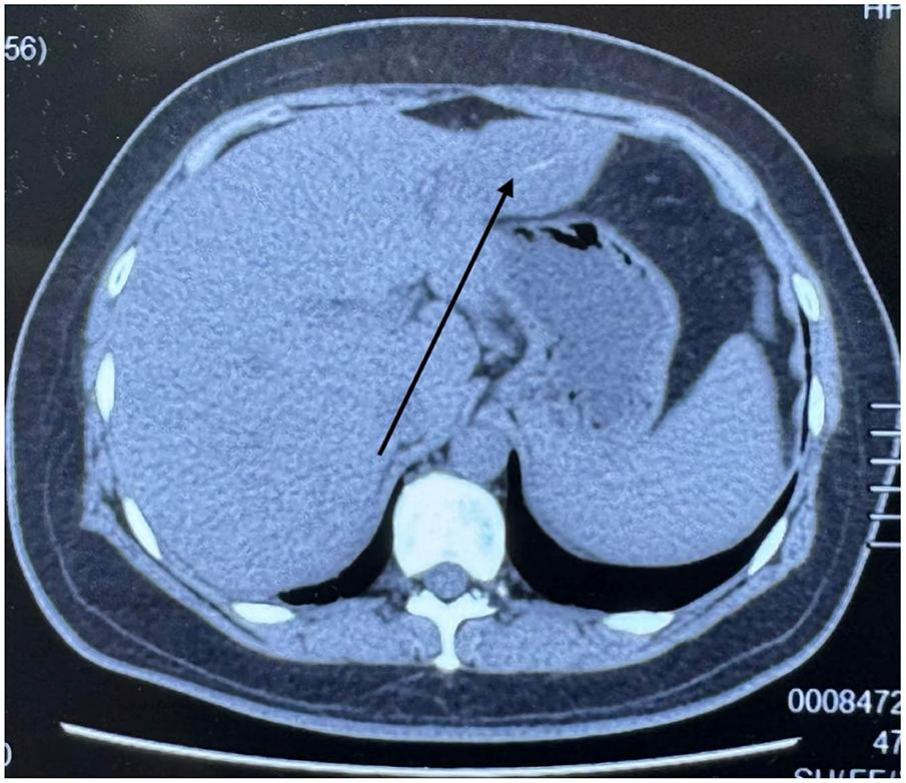
Non-contrast abdominal CT showing a left hepatic lobe abscess-like lesion with an intralesional linear hyperdense focus (arrow). Axial non-contrast CT demonstrates an ill-defined, round hypodense lesion in the left hepatic lobe. A linear hyperdense structure is visible within the lesion, raising suspicion for a migrated foreign body–associated infectious process. *Contrast-enhanced phase images were not available for review in this report.*

Laboratory tests, including liver biochemistry and serum tumor markers, were unremarkable, and hepatic reserve was preserved (Child–Pugh A, 5). The patient had leukocytosis with neutrophilia (WBC 13.3 × 10^9/L; neutrophils 87%) and an elevated C-reactive protein level (45.4 mg/L), while procalcitonin was normal (0.03 ng/mL). Non-contrast abdominal CT showed a left-lobe infectious-appearing lesion containing an intralesional linear hyperdense focus, raising suspicion for a migrated foreign body with pyogenic liver abscess formation (Figure 1). Contrast CT was not repeated to avoid delaying definitive management, as non-contrast CT already provided the key clue; no major vascular pedicle involvement was evident. After multidisciplinary discussion, the differential diagnosis included a typical pyogenic liver abscess and a necrotic/hemorrhagic hepatic lesion; however, the inflammatory markers and partial response to antibiotics favored an infectious etiology. Accordingly, foreign body–related PLA was strongly suspected preoperatively and was confirmed by intraoperative pus aspiration and toothpick retrieval, with supportive postoperative histopathology.

## Treatment and operative findings

After admission, empirical broad-spectrum antimicrobial therapy was initiated to cover Gram-negative organisms and anaerobes for 3 days (cefoperazone/sulbactam 3 g every 12 h plus ornidazole 1 g once daily). Given the CT suspicion of an intrahepatic foreign body and the potential risk of gastrointestinal perforation, percutaneous drainage was not pursued, and definitive surgical management was selected. Because foreign-body–related PLA often fails to resolve without complete foreign body removal and definitive treatment of the perforation tract, and because the lesion was localized to segments II/III with preserved liver function, laparoscopic left lateral sectionectomy was chosen to achieve reliable source control and reduce recurrence. A definite gastric wall defect was not identified preoperatively on non-contrast imaging; the perforation was confirmed intraoperatively during adhesiolysis.

On September 19, 2025, under general anesthesia, the patient underwent laparoscopic left lateral sectionectomy (segments II and III) with adhesiolysis, followed by laparoscopic repair of a gastric perforation. No significant ascites was present and no gross involvement of major vascular pedicles. Extensive adhesions were encountered between the omentum/bowel and the abdominal wall, as well as between the left lateral liver (segments II/III) and the abdominal wall. Dense adhesions were also noted between the anterior gastric wall and the falciform ligament. The foreign body was not visible externally on the liver surface; therefore, to avoid uncontrolled rupture and incomplete extraction, we proceeded with *en bloc* resection of the involved segments, and the toothpick was subsequently retrieved from the abscess cavity within the specimen.

**Figure 2:**
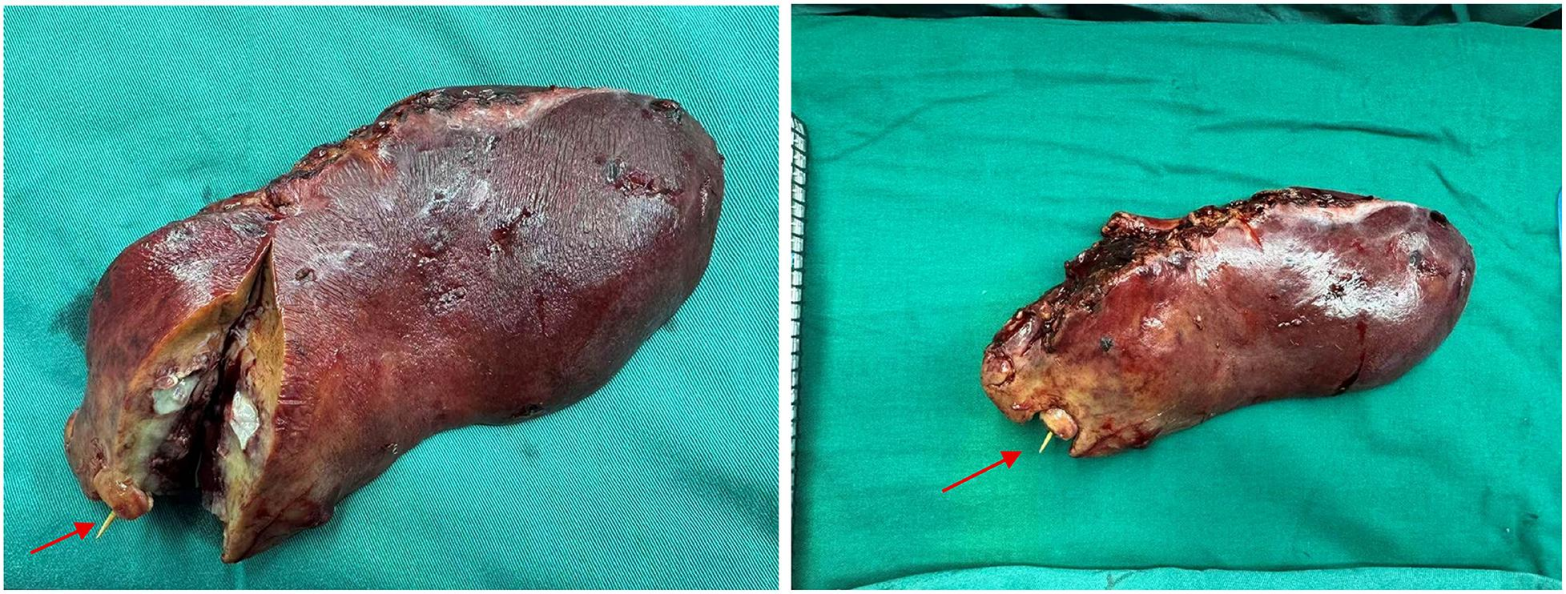
Gross specimen after laparoscopic left lateral sectionectomy showing an abscess cavity containing a toothpick (arrow). On sectioning, an abscess cavity with necrotic debris is identified, with a toothpick partially protruding.

**Figure 3:**
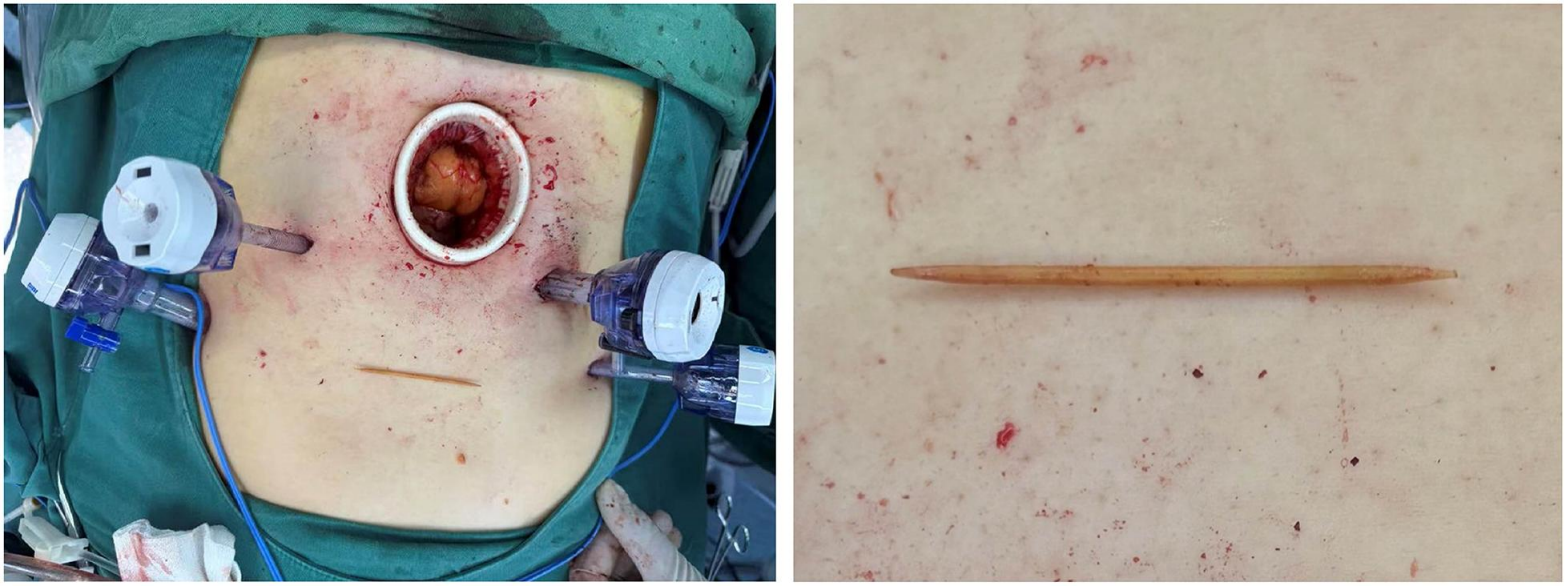
Intraoperative retrieval of an intact toothpick. The toothpick after retrieval, placed in the operative field during laparoscopy (Left). Gross appearance of the intact toothpick (Right).

During dissection, purulent material was noted oozing from the hepatic lesion, and approximately 100 mL of pus was aspirated. While separating the anterior gastric wall from the falciform ligament, a focal defect of the gastric wall was identified, consistent with gastric perforation; primary laparoscopic repair with reinforcement was immediately performed. Gross inspection of the resected specimen revealed gray-red/gray-white changes measuring approximately 6.0 × 3.8 cm. On sectioning, an abscess cavity containing purulent fluid and necrotic debris was identified (Figure 2), and an intact toothpick was retrieved from the cavity, confirming a foreign body–related infection (Figure 3). A single abdominal drain was placed in the right upper quadrant. Estimated blood loss was approximately 50 mL. The patient recovered uneventfully from anesthesia and returned to the ward in stable condition.

## Postoperative course and follow-up

Postoperatively, the abdominal drain output was clear, serosanguinous fluid, approximately 100–200 mL/day. Antibiotic therapy was continued for 1 week. The patient became afebrile on postoperative day (POD) 3. Repeat laboratory tests on POD 5 showed normalization of inflammatory markers. The abdominal drain was removed on POD 7, and the patient was discharged the same day.

Histopathological examination of the resected left lateral liver specimen demonstrated areas of hemorrhage with degenerative necrosis, surrounded by mixed acute and chronic inflammatory cell infiltration, consistent with pyogenic abscess formation. Mild steatosis was noted in the adjacent hepatic parenchyma. The foreign body was identified as a wooden toothpick.

At 4-month follow-up, the patient remained in good general condition without recurrent fever or abdominal pain. Follow-up non-contrast abdominopelvic CT demonstrated no residual abscess cavity (Table 1).

**Table 1:** Timeline of clinical events.

Date	Key event
2025-09-02	Onset of fever with upper abdominal pain.
2025-09-13 to 2025-09-18	Empirical antibiotic therapy initiated; diagnostic work-up completed; foreign body–related pyogenic liver abscess was suspected.
2025-09-19	Laparoscopic left lateral sectionectomy (segments II/III) with adhesiolysis and gastric perforation repair; an intact toothpick was retrieved from the abscess cavity; an abdominal drain was placed.
2025-09-20 to 2025-09-25	Continued antibiotics for 1 week; clinical symptoms improved and inflammatory markers normalized.
2025-09-26	Abdominal drain removed; patient discharged.
2025-09-27 to 2026-01-25	Follow-up showed good recovery without recurrent symptoms.

## Discussion

Foreign body–related pyogenic liver abscess (PLA) is a rare complication of gastrointestinal perforation and is often diagnosed late because most patients do not recall foreign-body ingestion. A systematic review of 178 cases reported a mean age of 56 years with male predominance (62%); abdominal pain (83%) and fever (78%) were the most common symptoms. Fish bones were the leading foreign body (51%), and *Streptococcus* species were frequently identified when cultures were available; importantly, most patients required surgery or combined interventional management in addition to antibiotics to achieve source control (6). Consequently, these cases are commonly misattributed to typical PLA or hepatic space-occupying lesions (e.g., tumors with necrosis/hemorrhage), delaying etiology-directed treatment (1, 2, 7).

Mechanistically, ingested sharp or rigid foreign bodies (e.g., toothpicks, fish bones, chicken bones) may perforate the gastric antrum/lesser curvature or duodenum, causing localized inflammation and occult perforation, and subsequently migrate into the liver parenchyma or subcapsular space to form an infectious focus. Owing to the anatomic proximity of the stomach/duodenum to the left hepatic lobe (especially segments II/III/IV), these abscesses preferentially involve the left lobe, as consistently reported in toothpick- and fish bone–related cases (8–10). In our patient, the lesion was located in the left lateral segments (II/III); intraoperatively, a focal defect of the anterior gastric wall was repaired and an intact toothpick was retrieved from the abscess cavity, confirming the “gastric perforation–migration–PLA” causal pathway.

Clinically, foreign body–related PLA may present with atypical or attenuated infectious features. Patients often have low-grade or intermittent fever, sometimes partially masked by prior antibiotic exposure, and may lack overt sepsis; abdominal pain or upper abdominal discomfort tends to predominate over chills or high fever (4, 6). In our case, the patient had only low-grade fever without rigors or jaundice, which could reduce clinical suspicion. However, epigastric tenderness and positive hepatic percussion pain suggested hepatic involvement. Laboratory testing showed leukocytosis with neutrophilia, supporting an active inflammatory state, whereas liver enzymes were largely normal—consistent with a localized abscess that may not cause prominent hepatocellular injury. Overall, these findings fit the common pattern of “localized infection with nonspecific systemic response.”

Imaging is pivotal for etiologic identification in foreign body–related PLA. Contrast-enhanced CT is generally considered the modality of choice, typically showing a hypodense hepatic lesion with rim enhancement; a linear or needle-like hyperdense focus within or adjacent to the abscess suggests a foreign body. Indirect signs may include focal thickening of the gastric antrum/duodenum, inflammatory fat stranding, and adhesions to the hepatic surface (8, 11–14). Importantly, wooden toothpicks and some fish bones may be inconspicuous under certain scanning conditions, increasing the risk of missed diagnosis. Accordingly, in cryptogenic, refractory, or recurrent liver abscess, careful CT review for linear densities and adjacent gastrointestinal inflammatory changes is warranted; thin-slice reconstruction, oral contrast, or repeat imaging may improve detection (4, 8).

In our case, the available non-contrast CT demonstrated an ill-defined, round hypodense lesion in the left hepatic lobe with an intralesional linear hyperdense focus, raising suspicion for a migrated foreign body. Unfortunately, the arterial/portal venous phase image series from the outside institution could not be retrieved for review; therefore, we did not base diagnostic reasoning on enhancement characteristics. The diagnosis was ultimately established by definitive source evidence—retrieval of an intact toothpick from the abscess cavity with concomitant gastric perforation repair—and histopathology consistent with abscess formation.

The cornerstone of management is antimicrobial therapy plus definitive source control. While standard PLA is often treated with antibiotics and percutaneous drainage when feasible, foreign body–related PLA may persist or recur if the foreign body remains, and can progress to severe complications such as portal vein thrombosis, peritonitis, or sepsis. Multiple toothpick-related reports emphasize that cure is unlikely without foreign body removal and treatment of the perforation tract (10–12). Therefore, when imaging suggests a foreign body, gastrointestinal perforation is suspected, or the abscess anatomy is unfavorable for percutaneous drainage, surgical (or combined interventional) management aimed at foreign body extraction and perforation repair is warranted (15). Laparoscopy can provide minimally invasive, direct-vision adhesiolysis and effective source control, particularly for superficial or localized left-lobe lesions (2, 5, 16). In our case, empirical broad-spectrum antibiotics covering Gram-negative and anaerobic organisms were followed by laparoscopic left lateral sectionectomy with concomitant gastric perforation repair, which confirmed the toothpick etiology and resulted in an uneventful recovery, supporting an etiology-directed source-control strategy when a migrated foreign body is suspected (6, 17).

This case has limitations. Blood cultures and abscess/pus cultures were not obtained, precluding pathogen identification and susceptibility-guided antimicrobial optimization. Nevertheless, the definitive intraoperative evidence (an intact toothpick within the abscess cavity and concomitant gastric perforation) provides a strong causal link, and the rapid normalization of inflammatory markers with no recurrence on follow-up supports the effectiveness of the source-control strategy.

## Conclusion

Foreign body–related pyogenic liver abscess is uncommon and may present with nonspecific symptoms, leading to delayed diagnosis. In patients with cryptogenic, refractory, or recurrent liver abscess—particularly when CT shows an abscess-like lesion with an intralesional linear hyperdense focus—migration of an ingested foreign body with occult gastrointestinal perforation should be suspected and prompt multidisciplinary evaluation is warranted. In left-lobe abscesses with a linear hyperdense focus, surgical exploration and etiology-directed source control should be prioritized, as antibiotics and percutaneous drainage alone may fail when the foreign body and perforation tract persist. This case underscores the clinical value of recognizing key imaging clues and implementing timely, etiology-directed management.

## Data Availability

The raw data supporting the conclusions of this article will be made available by the authors, without undue reservation.
